# Case Report: Favorable Response to the Tyrosine Kinase Inhibitor Apatinib in Recurrent Merkel Cell Carcinoma

**DOI:** 10.3389/fonc.2021.625360

**Published:** 2021-03-03

**Authors:** Yiyi Feng, Xin Song, Renbing Jia

**Affiliations:** Department of Ophthalmology, Ninth People's Hospital, Shanghai Jiao Tong University School of Medicine, Shanghai Key Laboratory of Orbital Diseases and Ocular Oncology, Shanghai, China

**Keywords:** Merkel cell carcinoma, tyrosine kinase inhibitor, apatinib, eyelid, targeted therapy

## Abstract

**Background:**

As angiogenesis is an essential step in tumor growth and metastasis, the tyrosine kinase inhibitor (TKI) apatinib has become a revolutionary anticancer therapy across various malignancies. However, its efficiency and safety in Merkel cell carcinoma (MCC) are uncertain.

**Case presentation:**

The current study described the case of a 91-year-old man who presented with a 3.2 × 3.0 × 2.2 cm rapidly growing, solitary tumor of the right lower eyelid. It was diagnosed as MCC pathologically. Twenty-seven days after the surgery, the patient returned to the hospital with recurrent MCC. Apatinib was then administered to this patient. The patient had a complete response (CR) to apatinib after 4.4 months of targeted therapy. Twenty-seven months of progression-free survival (PFS) was achieved with controllable treatment-related adverse events (AEs).

**Conclusion:**

Treatment with apatinib demonstrated clinical benefit in our patient with recurrent MCC, highlighting its potential utility in other MCC patients. Further clinical trials are needed to determine the efficacy and safety of apatinib in MCC patients.

## Introduction

Merkel cell carcinoma (MCC) is a rare but highly aggressive cutaneous malignancy with neuroendocrine features that has 33–46% mortality (1, 2). Heath M et al. (3) summarized the clinical features of MCC in an acronym: AEIOU—Asymptomatic/lack of tenderness, Expanding rapidly, Immune suppression, Older than age 50, and ultraviolet (UV)-exposed site on a person with fair skin. The incidence rate of MCC varies across the world, with approximately 2,488 cases per year diagnosed in the United States (4). Merkel cell polyomavirus (MCPyV) and UV exposure play a major role in the pathogenesis of MCC (2). The most common primary sites of MCC are head and neck (45%), and eyelid tumors represent only 2.5% of cases (5).

Wide excision of the tumor in combination with adjuvant radiation therapy to the primary site is the first-line strategy (6). Chemotherapy and immunotherapy can be used to treat metastatic or unresectable MCC (6). In immunotherapy, immune checkpoint inhibitors targeting programmed cell death protein 1 (PD-1) or its ligand (PD-L1) are the favored agents. In addition, as tumor angiogenesis is one of the features of cancer, the inhibition of vascular endothelial growth factor (VEGF) signaling pathway has become a revolutionary anticancer approach across various malignancies (7). However, its efficacy and safety in MCC patients are unknown. Here, we report an elderly male who developed MCC of the eyelid and was treated with apatinib, a small molecule inhibitor of vascular endothelial growth factor receptor-2 (VEGFR-2).

## Case Presentation

The study was carried out according to the principles of the Declaration of Helsinki; informed consent has been obtained from the patient.

**Figure 1:**
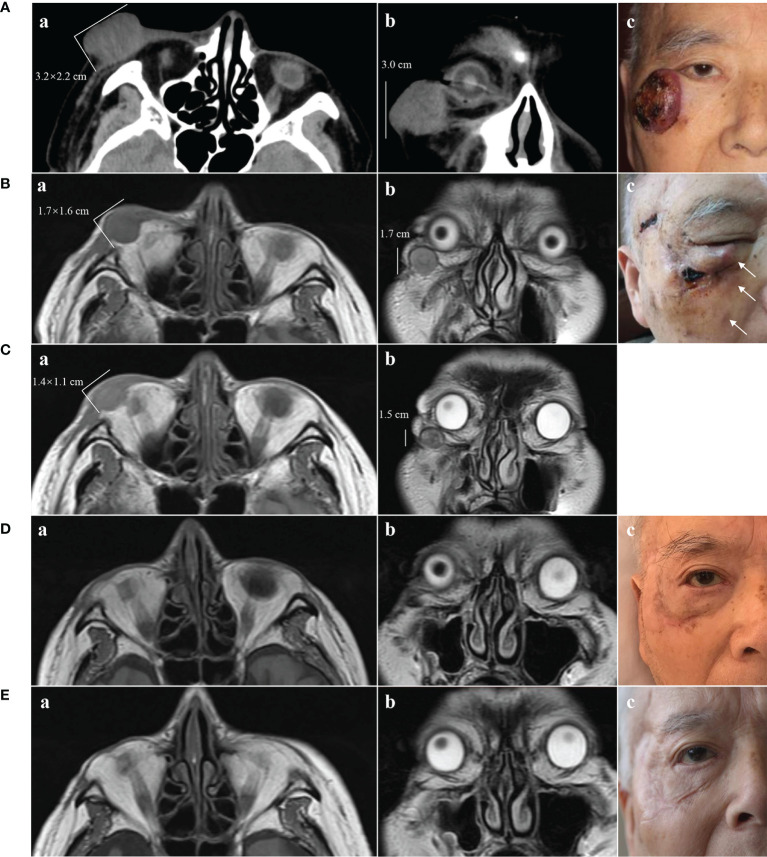
Clinical presentation of MCC and changes on imaging. **(A)** May 2018. **A**(a) and **A**(b) CT scans showing a tumor of 3.2 × 2.2 × 3.0 cm without destruction of bone. **A**(c) Solitary violet-colored nodule of the right lower eyelid with pigmentation and an irregular ulcer in the center. **(B)** June 2018. **B**(a) and **B**(b) MRI showing a tumor of 1.7 × 1.6 × 1.7 cm. **B**(c) Recurrence of the MCC. The white arrows show three hard, subcutaneous nodules. **(C)** July 2018, 2 weeks after treatment with apatinib. **C**(a) and **C**(b) MRI showing regression of the MCC (1.4 × 1.1 × 1.5 cm). **(D)** November 2018, 4 months after treatment with apatinib. **D**(a) and **D**(b) MRI showing MCC disappearance with a favorable response to apatinib. **D**(c) Only a scar with pigmentation was observed. **(E)** November 2019, 17 months after treatment with apatinib. **E**(a) and **E**(b) MRI showing no sign of recurrence. **E**(c) The pigmentation gradually subsided, leaving a pink scar.

**Figure 2:**
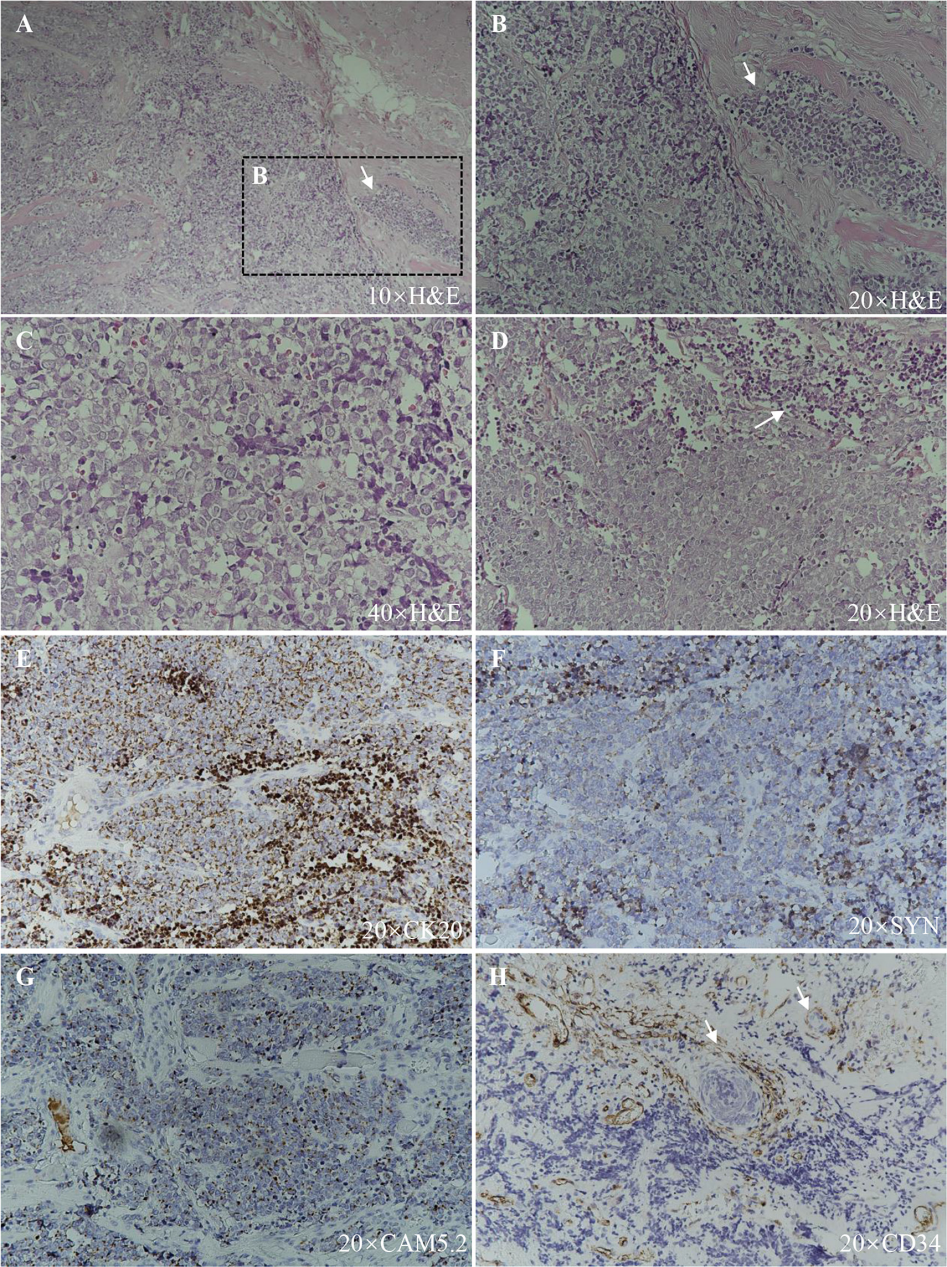
Histopathologic features. **(A)**, **(B)**, and **(C)** Hematoxylin–eosin staining, showing small, monomorphic, round-to-oval, low-differentiated cells with a vesicular nucleus and scant cytoplasm with muscle infiltration (arrow). **(D)** Hematoxylin–eosin staining, showing necrosis (arrow). **(E)** CK20 (+). **(F)** SYN (+). **(G)** CAM5.2 (+). **(H)** CD34 (+), blood vessels and tumor cells within them are indicated with arrows.

**Figure 3:**
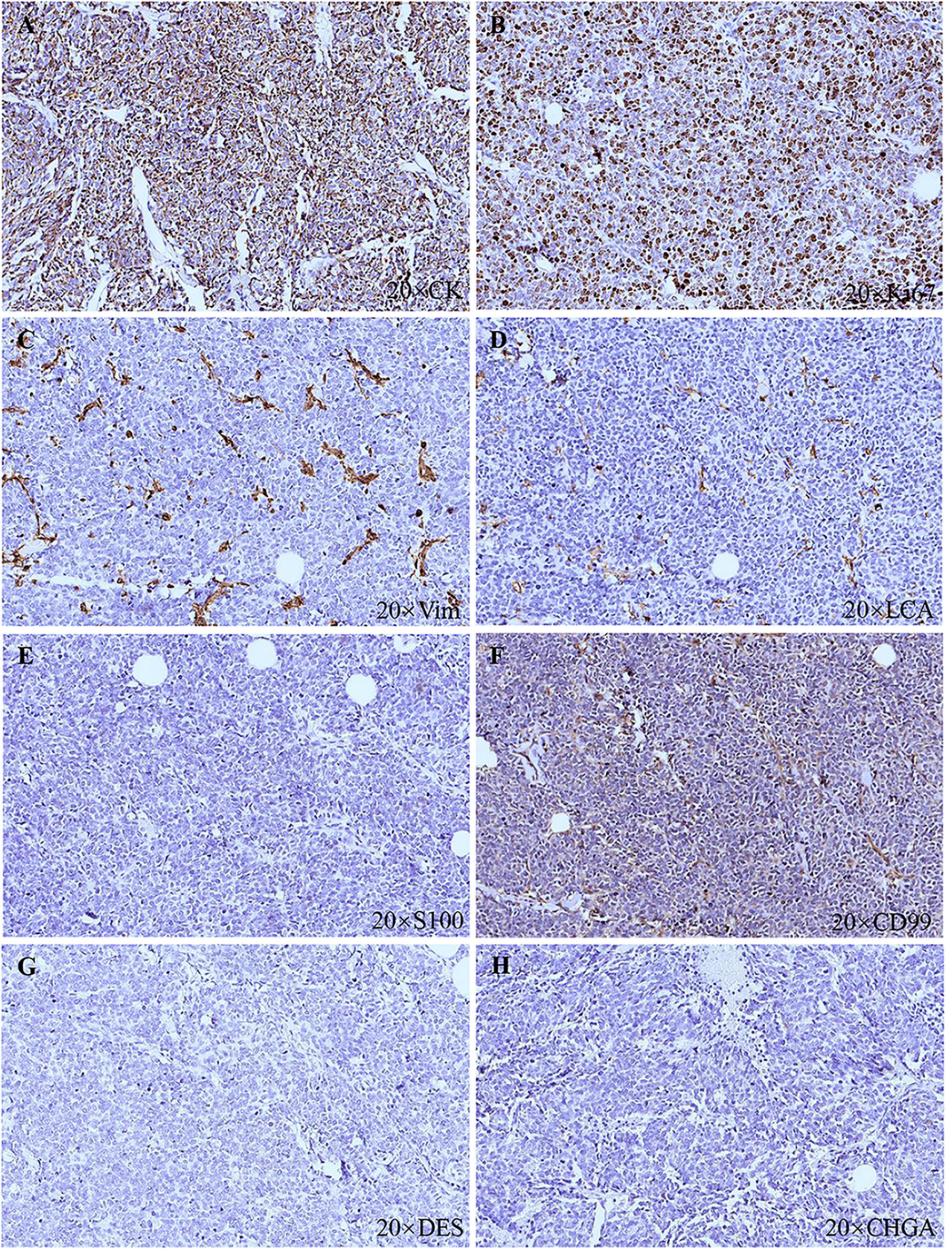
Histopathologic features. **(A)** CK (+). **(B)** Ki67 (80%+). **(C)** Vim (−). **(D)** LCA (−). **(E)** S100 (−). **(F)** CD99 (−). **(G)** DES (−). **(H)** CHGA (−).

A 91-year-old Chinese man presented in the Ninth People’s Hospital, Shanghai Jiao Tong University School of Medicine, on May 7, 2018, with a rapidly growing, solitary tumor of the right lower eyelid, which was initially noted in March 2018 without tenderness. Clinically, the tumor was a violet-colored nodule of 3.2 × 3.0 × 2.0 cm with pigmentation and an irregular ulcer in the center (**Figure 1A**). Fine needle aspiration biopsy was performed at Hua Shan Hospital, Fu Dan University, on April 17, 2018, which confirmed the diagnosis of MCC. The patient suffered from prostate cancer, hypertension, coronary heart disease (CHD), chronic cardiac insufficiency (NYHA, II–III) and chronic renal insufficiency and had received treatments of Enantone (3.75 mg, H, q4w), Adalat (30 mg, po, qd), Diovan (80 mg, po, qd), Furosemide (30 mg, po, qd) and spironolactone (20 mg, po, qd). He also had a history of pulmonary tuberculosis when he was young. Ultrasound and computed tomography (CT) were performed to clinically assess the tumor and cervical lymph nodes, and no signs of cervical lymph node metastasis were found. Clinical detection of lymph nodes or metastatic disease was performed *via* inspection and palpation, as the patient could not tolerate the long time required to complete the imaging examination. The physical examination was negative. After a multidisciplinary meeting, we decided to treat this patient with surgery, and sentinel lymph node biopsy (SLNB) was not considered due to the negative results of the imaging examination. Mohs micrographic surgery with a 1 cm excision margin was performed on May 10, 2018. The tumor had infiltrated the periosteum, and all the infiltrated soft tissue was removed together with the tumor. After confirmation of negative margins, reconstruction was performed. A rotation flap was designed to repair skin defects. Histologically small, monomorphic, round-to-oval, low-differentiated cells with a vesicular nucleus and scant cytoplasm were observed, which invaded the muscle, nerve and blood vessels (**Figure 2**). Necrosis was prominent (**Figure 2**). The immunohistochemistry results indicated the following patterns: CK (+), CK20 (+), SYN (+), CAM5.2 (+), CD34 (+), Ki67 (80%+), Vim (−), LCA (−), S100 (−), CD99 (−), DES (−), and CHGA (−) (**Figures 2**, **3**). According to the American Joint Committee on Cancer (AJCC) staging system, the final clinical diagnosis was MCC of the lower right eyelid, IIB. Postoperative radiotherapy was strongly recommended. However, the patient refused.

**Figure 4:**
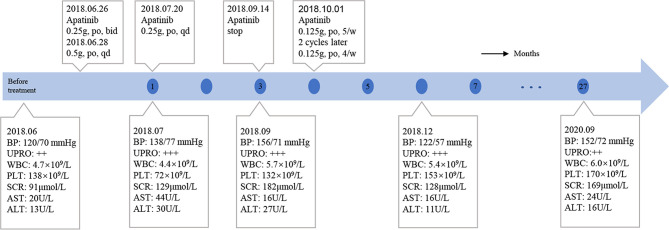
Time Course. Blue dots indicated passing months after the use of apatinib.

On June 6, 2018, several subcutaneous, hard nodules were observed at the primary site of surgery (**Figure 1B**). Chest CT, ultrasonography of the liver and kidneys, inspection and palpation of skin and lymph nodes ruled out distant metastasis. Considering the patient’s physical condition, surgery was abandoned after a multidisciplinary discussion. Ultimately, apatinib was used to treat MCC in this patient from June 26, 2018 (0.25 g, po, bid). As the treatment was well tolerated by the patient, two days later, we changed the dose of apatinib (0.5 g, po, qd). Blood pressure, routine blood tests, renal function, and liver function were carefully monitored (**Figure 4**). The MCC showed a strong response to apatinib, and the efficacy was significant (**Figure 1C**). However, on July 20, 2018, exacerbated proteinuria and thrombocytopenia led us to reduce the dose of apatinib (0.25 g, po, qd). The patient was treated with leucogen (20 mg, po, tid), and his thrombocytopenia resolved. On September 14, 2018, we stopped the use of apatinib due to a high serum creatinine level (182 μmol/L). Hand–foot syndrome also occurred. However, these treatment-related adverse events (AEs) were well controlled with symptomatic treatment. On October 1, 2018, after another multidisciplinary meeting, we restarted treatment (0.125 g, po, five times a week). After two cycles, we changed the dose of apatinib (0.125 g, po, four times a week) to a low maintenance dose. On November 9, 2018, 4.4 months after the first administration of apatinib, the patient had a complete response (CR) (**Figure 1D**). We continued administering low-dose apatinib for the treatment of MCC. In the following follow-up, the patient’s condition was stable (**Figure 1E**). Unfortunately, the patient died on October 4, 2020 due to heart failure and respiratory failure with no sign of recurrence or distant metastasis.

## Discussion

To the best of our knowledge, we report the first case of MCC of the eyelid treated with apatinib. As previously described, this case of MCC had a strong response to apatinib with few AEs. In addition, the effect was durable. Finally, the progression-free survival (PFS) of this patient was 27 months.

MCC is an aggressive skin cancer that is associated with exposure to UV radiation and MCPyV, with a median interval to recurrence of 8−9 months (8, 9). Wide excision is the first choice for the treatment of MCC. The National Comprehensive Cancer Network (NCCN) Merkel Cell Carcinoma Panel recommends adjuvant radiotherapy to the primary site for all patients with large primary tumors (≥1 cm) and risk factors such as lymphovascular invasion (LVI) or immunosuppression (6). Whether to apply radiotherapy to the draining nodal basin depends on the result of SLNB (negative or positive). Patients who do not undergo SLNB or LN dissection are also recommended to receive radiotherapy. The dosage of radiotherapy depends on the pathology of the resection margins and the result of SLNB. In this case, SLNB was not performed, and as infiltration of muscle, nerve and blood vessels was observed histologically, radiotherapy was recommended according to the NCCN MCC guidelines. However, the patient refused.

For unresectable MCC and metastatic MCC, systemic therapy is the choice, including chemotherapy and immunotherapy. The effect of chemotherapy varies from study to study. The objective response rate (ORR) for first-line chemotherapy ranged from 29.4 to 55%, and the durability of response (DOR) was 2.8−6.7 months (10–12). In patients who received one or more prior lines of chemotherapy, the ORR was 10.3−28.6%, and the DOR was 1.9−3.4 months (10–12). The PFS was 3.1−4.6 months for those patients receiving first-line chemotherapy and as low as 2−3 months in patients who received one or more prior lines of chemotherapy. In addition to the low response rates and limited durability, chemotherapy may cause toxicity, and it is not a suitable choice for elderly people with many underlying diseases, who have a higher risk of developing AEs. In this case report, the patient was 91 years old and had multiple underlying diseases, and chemotherapy was not chosen to treat the recurrent MCC. Regarding immunotherapy, PD-1 and PD-L1 are immune checkpoint molecules that control tumor growth. Immune checkpoint inhibitors (ICIs), such as avelumab (anti-PD-L1 antibody), nivolumab (anti-PD-1 antibody), and pembrolizumab (anti-PD-1 antibody), are used for the treatment of MCC. Some clinical trials of therapeutic antibodies against PD-1 or PD-L1 have showed high and durable response rates (2, 13, 14). The results of a multicenter, phase II trial of first-line use of pembrolizumab in patients with unresectable advanced MCC demonstrated an ORR of 56% and a DOR of 2.2−9.7 months (NCT02267603) (13). An international, multicenter clinical trial of first-line use of avelumab in metastatic MCC indicated an ORR of 62.1% and a DOR of at least 3 months (93%) (14).

Angiogenesis is a necessary step in tumor growth and metastasis. Among angiogenic factors, VEGF is the most potent. There are three molecular subtypes of the VEGF receptor (VEGFR), including VEGFR-1, VEGFR-2, and VEGFR-3. These receptors are type II transmembrane proteins characterized by tyrosine kinase (TK) activity (7). Among them, VEGFR-2 is the principal subtype of VEGF-induced angiogenic signaling (7). Several studies showed that VEGF and VEGFR-2 were overexpressed in MCC (15–17), and the upregulation of VEGF was associated with aggressive tumor behavior (15). Hence, VEGF and VEGFR can be potential targets for targeted therapy and have attracted increasing attention for the treatment of MCC. One study showed the efficacy of an anti-VEGF antibody (bevacizumab) in MCC in a mouse model (18), however, it has not yet been studied in clinical trials. Tyrosine kinase inhibitors (TKIs) are another potential choice, and their efficacy in other malignancies is impressive. However, little is known about their clinical benefit in MCC. To date, only one clinical trial of TKIs (NCT02036476) has been registered (19); however, due to toxicity and a lack of response, it was closed prematurely. In addition, some case reports have demonstrated the efficiency of TKIs, such as pazopanib and cabozantinib (20, 21). In this case report, we report impressive tumor regression in a patient with recurrent unresectable MCC during treatment with apatinib. Apatinib is a new inhibitor of VEGFR-2 TK activity targeting the intracellular ATP binding site of the receptor (22). The most frequent adverse events of apatinib included hypertension, proteinuria, and hand–foot syndrome, which were also observed in our patient. However, they could be controlled clinically. Our patient had an impressive response to apatinib, and he tolerated the treatment well with controllable AEs.

In conclusion, apatinib had a favorable effect with great durability in this patient, highlighting its potential utility in other MCC patients, especially those who cannot tolerate chemotherapy and those who do not respond to immunotherapy. Further clinical trials are needed to determine the efficacy and safety of apatinib in MCC patients.

## Data Availability Statement

The original contributions presented in the study are included in the article/supplementary material. Further inquiries can be directed to the corresponding authors.

## Ethics Statement

Written informed consent was obtained from the individual(s) for the publication of any potentially identifiable images or data included in this article.

## Author Contributions

XS and RJ provided direction and guidance throughout the preparation of this manuscript. XS and YF extracted all data. YF drafted the paper. All authors contributed to the article and approved the submitted version.

## Funding

This work was supported by The National Natural Science Foundation of China (Grant Nos. 81570884 and 81770961) and The Science and Technology Commission of Shanghai (Grant No.17DZ2260100).

## Conflict of Interest

The authors declare that the research was conducted in the absence of any commercial or financial relationships that could be construed as a potential conflict of interest.

## References

[1] Schadendorf D Lebbé C Zur Hausen A Avril MF Hariharan S Bharmal M. Merkel cell carcinoma: Epidemiology, prognosis, therapy and unmet medical needs. Eur J Cancer (2017) 71:53–69. 10.1016/j.ejca.2016.10.022 27984768

[2] Becker JC Stang A DeCaprio JA Cerroni L Lebbé C Veness M. Merkel cell carcinoma. Nat Rev Dis Primers (2017) 3:17077. 10.1038/nrdp.2017.77 29072302 PMC6054450

[3] Heath M Jaimes N Lemos B Mostaghimi A Wang LC Peñas PF. Clinical characteristics of Merkel cell carcinoma at diagnosis in 195 patients: the AEIOU features. J Am Acad Dermatol (2008) 58(3):375–81. 10.1016/j.jaad.2007.11.020 PMC2335370 18280333

[4] Paulson KG Park SY Vandeven NA Lachance K Thomas H Chapuis AG. Merkel cell carcinoma: Current US incidence and projected increases based on changing demographics. J Am Acad Dermatol (2018) 78(3):457–63.e2. 10.1016/j.jaad.2017.10.028 29102486 PMC5815902

[5] Lemos BD Storer BE Iyer JG Phillips JL Bichakjian CK Fang LC. Pathologic nodal evaluation improves prognostic accuracy in Merkel cell carcinoma: analysis of 5823 cases as the basis of the first consensus staging system. J Am Acad Dermatol (2010) 63(5):751–61. 10.1016/j.jaad.2010.02.056 PMC2956767 20646783

[6] Bichakjian CK Olencki T Aasi SZ Alam M Andersen JS Blitzblau R. Merkel Cell Carcinoma, Version 1.2018, NCCN Clinical Practice Guidelines in Oncology. J Natl Compr Canc Netw (2018) 16(6):742–74. 10.6004/jnccn.2018.0055 PMC9440150 29891526

[7] Zhang H. Apatinib for molecular targeted therapy in tumor. Drug Des Devel Ther (2015) 9:6075–81. 10.2147/DDDT.S97235 PMC4654530 26622168

[8] Santamaria-Barria JA Boland GM Yeap BY Nardi V Dias-Santagata D Cusack JC. Merkel cell carcinoma: 30-year experience from a single institution. Ann Surg Oncol (2013) 20(4):1365–73. 10.1245/s10434-012-2779-3 23208132

[9] Hui AC Stillie AL Seel M Ainslie J. Merkel cell carcinoma: 27-year experience at the Peter MacCallum Cancer Centre. Int J Radiat Oncol Biol Phys (2011) 80(5):1430–5. 10.1016/j.ijrobp.2010.04.061 20708847

[10] Iyer JG Blom A Doumani R Lewis C Tarabadkar ES Anderson A. Response rates and durability of chemotherapy among 62 patients with metastatic Merkel cell carcinoma. Cancer Med (2016) 5(9):2294–301. 10.1002/cam4.815 PMC5055152 27431483

[11] Becker JC Lorenz E Ugurel S Eigentler TK Kiecker F Pfohler C. Evaluation of real-world treatment outcomes in patients with distant metastatic Merkel cell carcinoma following second-line chemotherapy in Europe. Oncotarget (2017) 8(45):79731–41. 10.18632/oncotarget.19218 PMC5668086 29108353

[12] Cowey CL Mahnke L Espirito J Helwig C Oksen D Bharmal M. Real-world treatment outcomes in patients with metastatic Merkel cell carcinoma treated with chemotherapy in the USA. Future Oncol (London England) (2017) 13(19):1699–710. 10.2217/fon-2017-0187 28605939

[13] Nghiem PT Bhatia S Lipson EJ Kudchadkar RR Miller NJ Annamalai L. PD-1 Blockade with Pembrolizumab in Advanced Merkel-Cell Carcinoma. N Engl J Med (2016) 374(26):2542–52. 10.1056/NEJMoa1603702 PMC4927341 27093365

[14] D’Angelo SP Russell J Lebbe C Chmielowski B Gambichler T Grob JJ. Efficacy and safety of first-line avelumab treatment in patients with stage IV metastatic Merkel cell carcinoma: a preplanned interim analysis of a clinical trial. JAMA Oncol (2018) 4(9):e180077. 10.1001/jamaoncol.2018.0077 29566106 PMC5885245

[15] Fernández-Figueras MT Puig L Musulén E Gilaberte M Lerma E Serrano S. Expression profiles associated with aggressive behavior in Merkel cell carcinoma. Mod Pathol (2007) 20(1):90–101. 10.1038/modpathol.3800717 17115023

[16] Brunner M Thurnher D Pammer J Geleff S Heiduschka G Reinisch CM. Expression of VEGF-A/C, VEGF-R2, PDGF-alpha/beta, c-kit, EGFR, Her-2/Neu, Mcl-1 and Bmi-1 in Merkel cell carcinoma. Mod Pathol (2008) 21(7):876–84. 10.1038/modpathol.2008.63 18408656

[17] Kukko H Koljonen V Lassus P Tukiainen E Haglund C Böhling T. Expression of vascular endothelial growth factor receptor-2 in Merkel cell carcinoma. Anticancer Res (2007) 27(4C):2587–9. 17695419

[18] Kervarrec T Gaboriaud P Tallet A Leblond V Arnold F Berthon P. VEGF-A Inhibition as a Potential Therapeutic Approach in Merkel Cell Carcinoma. J Invest Dermatol (2019) 139(3):736–9. 10.1016/j.jid.2018.08.029 30359576

[19] Rabinowits G Lezcano C Catalano PJ McHugh P Becker H Reilly MM. Cabozantinib in Patients with Advanced Merkel Cell Carcinoma. Oncologist (2018) 23(7):814–21. 10.1634/theoncologist.2017-0552 PMC6058327 29445030

[20] Davids MS Davids M Charlton A Ng SS Chong ML Laubscher K. Response to a novel multitargeted tyrosine kinase inhibitor pazopanib in metastatic Merkel cell carcinoma. J Clin Oncol (2009) 27(26):e97–100. 10.1200/JCO.2009.21.8149 19564526

[21] Tarabadkar ES Thomas H Blom A Parvathaneni U Olencki T Nghiem P. Clinical Benefit from Tyrosine Kinase Inhibitors in Metastatic Merkel Cell Carcinoma: A Case Series of 5 Patients. Am J Case Rep (2018) 19:505–11. 10.12659/AJCR.908649 PMC5952731 29706615

[22] Roviello G Ravelli A Polom K Petrioli R Marano L Marrelli D. Apatinib: A novel receptor tyrosine kinase inhibitor for the treatment of gastric cancer. Cancer Lett (2016) 372(2):187–91. 10.1016/j.canlet.2016.01.014 26797419

